# Supplementary material for: A set of multi-entry identification keys to African frugivorous flies (Diptera, Tephritidae)
Source: Zookeys. 2014 Jul 24;(428):97–108. doi: 10.3897/zookeys.428.7366 (PMC4143993; doi:10.3897/zookeys.428.7366)
Supplement: Supplementary material 4 — Key to Capparimyia [file zookeys-428-097-s004.zip › SF4_ZooKeys_key to Capparimyia/lucid_player/help/help/whgdata/whlstt2.htm]

Content


Help for the Lucid3 Player  
 What does the Lucid3 Player do?  
 Registration and Activation  
 The Applet Player and Application Player  
 System Requirements  
 Using the Lucid3 Player  
    Opening a Key  
    About Entities and Features  
    Trees and Lists  
    Collapsing and expanding tree items  
    Finding features, states and entities  
    Choosing states from multistate features  
    Choosing numeric features  
    Un-choosing states  
    Finding the best feature - The Best functions  
    Pruning the feature tree - The Prune Redundants function  
    Manually discarding and restoring Entities  
    Getting an Entities score value  
    Shortcuts to an answer - The Shortcut function  
    Using subsets of features and entities  
    Comparing entities - The Differences function  
    Multimedia in the Lucid3 Player  
    Feature Gallery View  
    Restarting the key  
    Configuring the Player  
 The Lucid Player�s Menu and Button Bar  
 About the Lucid Family of Products  
 About the Lucid3 Builder
